# Complex Systems Biology Approach in Connecting PI3K-Akt and NF-κB Pathways in Prostate Cancer

**DOI:** 10.3390/cells8030201

**Published:** 2019-02-26

**Authors:** Eswar Shankar, Michael C. Weis, Jayant Avva, Sanjeev Shukla, Meenakshi Shukla, Sree N. Sreenath, Sanjay Gupta

**Affiliations:** 1 Department of Urology, School of Medicine, Case Western Reserve University, Cleveland, OH 44106, USA; exs334@case.edu (E.S.); sanjeev.shukla@case.edu (S.S.); mxs276@case.edu (M.S.); 2 The Urology Institute, University Hospitals Cleveland Medical Center, Cleveland, OH 44106, USA; 3 Department of Electrical Engineering and Computer Science, School of Engineering, Case Western Reserve University, Cleveland, OH 44106, USA; mcw16@case.edu (M.C.W.); jxa86@case.edu (J.A.); nxs6@case.edu (S.N.S.); 4 Department of Urology, Louis Stokes Cleveland Veterans Affairs Medical Center, Cleveland, OH 44106, USA; 5 Department of Nutrition, Case Western Reserve University, Cleveland, OH 44106, USA; 6 Division of General Medical Sciences, Case Comprehensive Cancer Center, Cleveland, OH 44106, USA

**Keywords:** NF-κB, PI3K/Akt, systems biology

## Abstract

Phosphatidylinositol 3′-OH kinase (PI3K)-Akt and transcription factor NF-κB are important molecules involved in the regulation of cell proliferation, apoptosis, and oncogenesis. Both PI3K-Akt and Nuclear Factor-kappaB (NF-κB) are involved in the development and progression of prostate cancer, however, the crosstalk and molecules connecting these pathway remains unclear. A multilevel system representation of the PI3K-Akt and NF-κB pathways was constructed to determine which signaling components contribute to adaptive behavior and coordination. In silico experiments conducted using PI3K-Akt and NF-κB, mathematical models were modularized using biological functionality and were validated using a cell culture system. Our analysis demonstrates that a component representing the IκB kinase (IKK) complex can coordinate these two pathways. It is expected that interruption of this molecule could represent a potential therapeutic target for prostate cancer.

## 1. Introduction

Prostate cancer remains the third most common cancer worldwide with over one million new cases detected annually; it is the second leading cause of cancer-related deaths in men in the United States [1,2]. According to an estimate by the American Cancer Society, approximately 174,650 new prostate cancer cases will be diagnosed and 31,620 deaths will occur in the United States in 2019 [3]. Prostate cancer is a heterogeneous disease and often remains indolent where patients remain asymptomatic for years. It has been difficult, however, to discriminate between treatable and non-treatable tumors as concerns have been raised of increased over-diagnosis and unnecessary treatment [4]. At present, prognosis and treatment stratification is based on clinical stage, histologic evaluation with a Gleason grade as a measure of tumor differentiation, and serum prostate-specific antigen (PSA) levels, which do not accurately predict clinical outcome [4,5]. Hence, there is an urgent need to identify precise targets to determine appropriate treatments.

Several clinical and experimental studies have established that constitutive activation of the phosphatidylinositol 3′-OH kinase (PI3K)-Akt pathway is an essential step towards initiation and maintenance of various human malignancies, including prostate cancer [6,7,8]. The PI3K-Akt signaling pathway regulates several physiological functions including cell proliferation, survival, growth, and motility as these processes are critical for carcinogenesis [9,10]. PI3K is a heterodimeric protein and major signaling component downstream of growth factor receptor tyrosine kinases, consisting of a catalytic subunit (p110α/β/γ/δ) and a regulatory subunit (p85α/β) upregulated by several different mechanisms [10]. Following activation by various internal and external factors, PI3K is recruited to the plasma membrane, catalyzing the conversion of membrane phosphoinositide 4,5-biphosphate (PIP2) in the D3 position to generate phosphoinositide 3,4,5-triphosphate (PIP3). Accrual of PIP3 creates a docking site for Akt at the plasma membrane, inducing a conformational change, exposing the critical residues Thr308/309 and Ser473/474 to phosphorylation by phosphotidylinositol-dependent kinase 1/2 [10,11]. Activation of PI3K-Akt is counterbalanced by the action of the phosphatase and tensin homolog (PTEN), a lipid phosphatase and tumor suppressor that dephosphorylates PIP3 back to PIP2, controlling the activation of Akt. PTEN is frequently lost in several human malignancies including glioblastomas and endometrial, melanoma, breast, and prostate cancer [12,13,14]. The PI3K-Akt pathway is a key regulator of cell survival through multiple downstream targets including the forkhead transcription factor (FOXO) family viz. AFX, FKHR, and FKHRL1, which are known to mediate apoptosis by activating the transcription of pro-apoptotic genes such as *FasL* and *Bim* [15]. Phosphorylation of FOXO members by Akt results in their cytoplasmic retention by interaction with 14-3-3 proteins, thereby sequestering them from their gene targets. (Figure S1) [16]. 

Constitutive activation of Nuclear Factor-kappaB (NF-κB) family members have been shown in clinical specimens, human derived cell lines, and in vivo models of prostate cancer [17,18]. NF-κB is a widely studied dimeric transcription factor induced by several stimuli including inflammatory cytokines, lipopolysaccharides, and others [19]. The five members that constitute the mammalian NF-κB family are RelA/p65, c-Rel, p50/NF-κB1, p52/NF-κB2, and RelB. NF-κB activation is controlled by the release from the IκB inhibitory proteins, which maintain NF-κB generally in the cytoplasm. Following stimulus, the IκB kinase (IKK) complex is activated and phosphorylates IκB proteins leading to their ubiquitination and subsequent proteasome-dependent degradation; NF-κB is released and translocates in the nucleus, binding with specific gene regulatory regions controlling gene expression. The IKK complex consists of two highly conserved catalytic subunits viz. IKKα and IKKβ, of which IKKβ appears to be the dominant kinase controlling IκB phosphorylation. Activation of NF-κB results in the induction of a variety of genes influencing cellular proliferation, inflammation, and adhesion [20,21]. A genome-wide association study and other gene expression studies have linked NF-κB-associated pathways to prostate cancer progression [22]. Within NF-κB itself, p100 and p105 can mediate interactions with NF-κB subunits that can also function as IκB proteins, activating or contributing to deregulation of the pathway (Figure S2) [20].

Among several identified pathways, signaling between NF-κB and PI3K-Akt has been pronounced as having a critical role in prostate cancer progression [23]. For example, the two PI3K inhibitors, LY294002 and Wortmannin, block the interleukin (IL)-1-induced increase in the DNA binding activity of NF-κB [24]. In addition, under some conditions, Akt can promote cell survival by indirectly activating the pro-survival transcription factor NF-κB through the phosphorylation of IKK [25]. Studies from our laboratory have demonstrated constitutive activation of NF-κB/p65 and Akt kinase (Ser473) during prostate cancer progression by utilizing clinical specimens and cell culture models [26,27]. We further demonstrated the involvement of these molecules in prostate cancer using an autochthonous transgenic mouse model [28]. However, the functional relationship between these pathways and their mode of interaction during prostate cancer progression have not been fully specified. Cancer-system biology requires the use of individual disciplines and data types, integrating experimental and computational approaches, to systematically study cancer [29,30]. In this study, we employ a complex-systems biology approach focused on a multilevel hierarchical paradigm to search for convergence of PI3K-Akt and NF-κB signaling pathways in prostate cancer. 

## 2. Materials and Methods

### 2.1. Computational Modeling and Simulations

A mathematical model was constructed based on mass action law and Michaelis–Menten approximation yielding a nonlinear ordinary differential equation (ODE), which was structurally calibrated and validated based on published experimental data [31,32,33]. The mathematical model in a general form was generated.

$$\begin{array}{l} \dot{x}\;=\;f_{1}(x,k)\;+\;f_{2}(x,k)+g(x,k)u,\\ y\;=\;h(x)\\ f_{1}(x,k)\;=\;S_{MM}v,\\ f_{2}(x,k)+g(x,k)u\;=\;(S_{Ma,out}-S_{Ma,in})diag(k)\exp(S_{Ma,in}{}^{T}\log(x))[(1+cu)] \end{array}$$

where *x* = state variable, or signal concentration; *h*(*x*) = variable relative value from Western blot; *y* = output-concentration measures; SMM = Michaelis–Menten stoichiometry matrix; k = rate constant or parameters; v = Michaelis–Menten reaction rates; u = input to the system; SMa, in = Input mass action stoichiometry matrix; c = matrix of constant multipliers; SMa, out = Output mass action stoichiometry matrix.

The model and its initial parameterization was assembled by adopting various existing models [34,35]. The kinetic parameters and initial molecular concentrations in this model were taken from published literature or derived from basic physicochemical quantities. Computational simulations were performed using a 2.7 GHz Pentium 4 PC, and the ODE solver (ode15s function) Matlab R14 (MathWorks, Inc., Natick, MA, USA) was used to solve the differential equations.

### 2.2. Cells and Treatments

RWPE1 cells were obtained from American Type Culture Collection. The cells were cultured in Keratinocyte Serum Free Medium, supplemented with 0.05 mg/mL bovine pituitary extract and 5 ng/mL epidermal growth factor (EGF) (Gibco Laboratories, Maryland, MD, USA). RWPE1 cells are established from epithelial cells derived from the peripheral zone of a histologically normal adult human prostate after transfection with a single copy of the human papilloma virus 18. These cells express cytokeratins 8 and 18, which are characteristic of luminal prostatic epithelial cells. Upon growth stimulation with synthetic androgens or stimulation by EGF, RWPE1 cells express prostate-specific antigens (PSA) and androgen receptors and are non-tumorigenic in mice [36]. For transient knockdown of Akt, dominant negative Akt (Akt DN) and empty vector were purchased from Upstate Cell Signaling (Lake Placid, NY, USA). Cells were transiently transfected using Lipofectamine™ 2000 reagent (Invitrogen Corporation, Carlsbad, CA, USA) with the vector in serum-free medium. Later, these cells were switched to complete-cell culture medium. In another experiment, 60% confluent cell cultures were switched to serum-free medium for 16 h, and then treated with specified doses of PI3K inhibitor LY294002 in complete-cell culture medium for 8 h. Cells from all the groups were photographed using light microscopy.

### 2.3. Cell Proliferation Assay

Cell proliferation was determined by measuring 3-(4,5-dimethylthiazol-2-yl)-2,5-diphenyltetrazolium bromide (MTT) (Sigma) colorimetric dye reduction. RWPE1 cells were cultured in 96-well plates at a cell density of 1 × 10^3^/well in complete Keratinocyte-SFM medium and allowed to incubate at 37 °C in a 5% CO_2_ environment. The cells were treated with various concentrations of EGF at various doses and times as indicated. Then, 10 μL of MTT reagent (5 mg/mL) was added to each well, the plates were incubated for 3 h at 37 °C, and the crystals were dissolved by using a solubilization solution. The absorbance was measured at 570 nm using a Bio-Rad plate reader. The percentage of cell proliferation was determined relative to the control.

### 2.4. PSA Measurements

Quantitative measurement of total PSA in cell culture media was performed using reagents and protocol of DSL-9700 Active PSA Coated-Tube IRMA kit provided by Diagnostic Systems Laboratories (Webster, TX, USA).

### 2.5. Western Blotting

For immunoblot analysis, 40 µg protein was resolved using 4–20% polyacrylamide gels (Novex, Carlsbad, CA, USA) and transferred to a nitrocellulose membrane. The blot was blocked in blocking buffer (5% nonfat dry milk/1% Tween 20; in 20 mM TBS, pH 7.6) for 2 h at room temperature, incubated with a primary antibody in blocking buffer for 2 h at room temperature or overnight at 40 °C, incubated with an appropriate secondary antibody conjugated with horseradish peroxidase (Amersham-Pharmacia, Piscataway, NJ, USA), detected by ECL-chemiluminescence, then underwent autoradiography using XAR-5 film (Eastman Kodak, Rochester, NY, USA). For equal loading of proteins, the membrane was probed with appropriate loading control. The antibodies used were anti-IKKα (Cat#2682) and anti-p-IKKα/β (Ser181/182; Cat#2697) from Cell Signaling Technology, Danvers, MA and Anti-Akt (Cat#sc-8312), anti-p-Akt (Ser473; Cat#sc-7985), anti-IκBα (Cat#sc-1643), anti-p-IκBα (Ser32/36; Cat#sc-8404), anti-PCNA (Cat#sc-56), and anti-β-Actin (Cat#sc-47778) from Santa Cruz Biotechnology, Santa Cruz, CA, USA. Nuclear lysates were prepared for estimation of NF-κB activation using anti-NF-κB/p65 antibody (Cat#sc-8008) procured from Santa Cruz as previously described [26].

### 2.6. Statistical Analysis

The significance between the control and treated groups were determined by the Student’s ‘*t*’ test and *p* values less than 0.05 were taken as significant.

## 3. Results

### 3.1. Pathway Modeling

**Figure 1:**
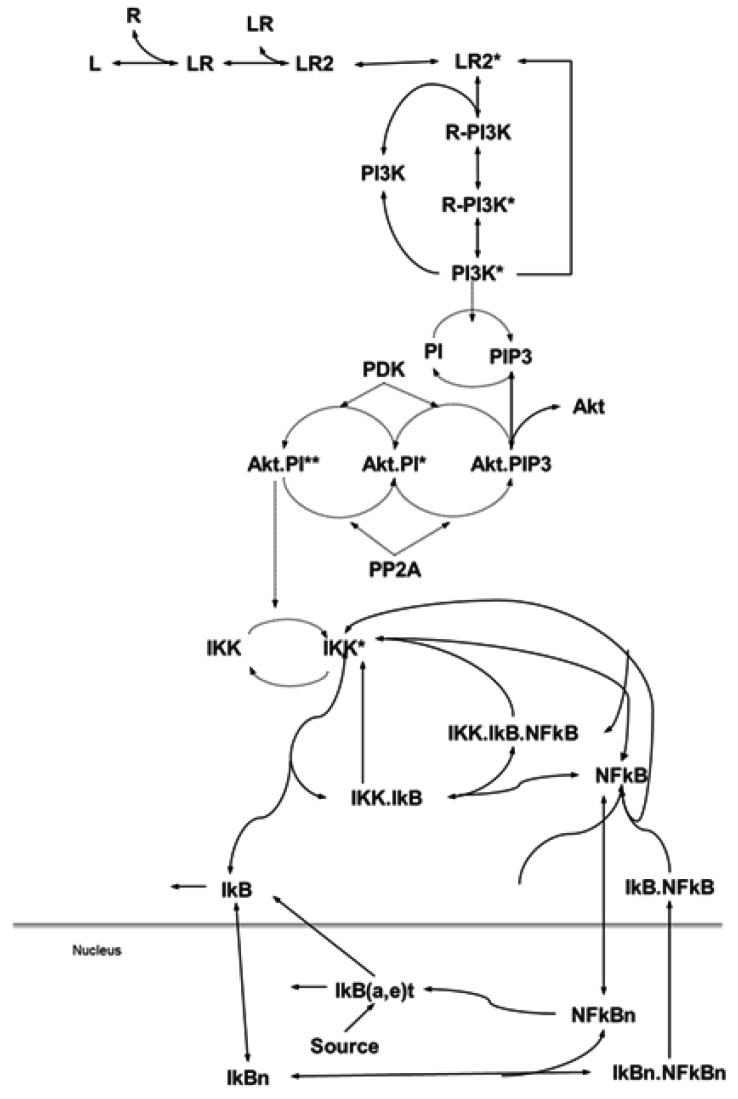
Biochemical reactions of the phosphatidylinositol 3′-OH kinase (PI3K)-Akt and Nuclear Factor-kappaB (NF-κB) pathway and its modularization. Modularization is based on the biological functionality and contiguity, where modeling biochemical reactions of the pathways via mass action law and Michaelis–Menten approximation (receptor) yields a nonlinear ordinary differential equation (ODE) model. Details are provided in the Materials and Methods section.

Firstly, we present an ordinary differential equation (ODE)-based mathematical model of an EGF-induced signaling network, which involves EGF-driving of a proliferation/pro-survival signaling pathway, PI3K/Akt, and its connection with NF-κB. As shown in Figure 1, the portions of the PI3K/Akt and NF-κB pathways in this model were established based on published signaling pathways [28,29,30,31]. The model comprises 96 biochemical reactions converted into 42 differential equations based on Michaelis–Menten approximations for basic enzymatic reactions and the Law of Mass Action for the rest of the reactions. The activation of IKK by Akt and its calibration was conducted through extensive simulations (>7000), categorizing the resulting NF-κB responses to Akt, and matching them with the published results. This exercise resulted in a complete model of EGF activated PI3K-Akt and NF-κB signaling systems that accurately reflects available data in the literature for validation.

### 3.2. Integration for Pathway Component Predictions

**Figure 2:**
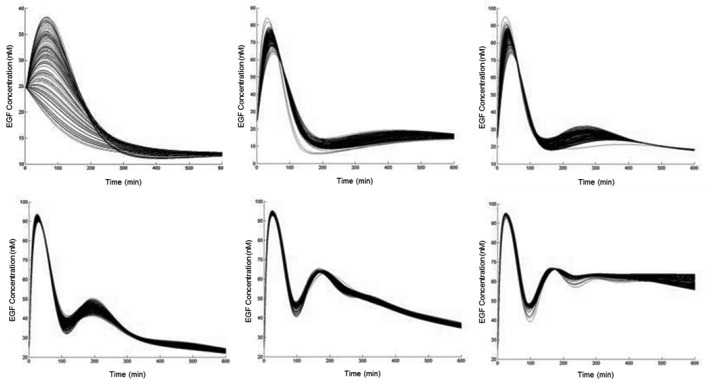
Categories of responses by epidermal growth factor (EGF)-mediated NF-κB activation. Time-courses of NF-κB in response to 10 nM of EGF over 10 h. Parameters for the biochemical reaction governing the action of IKK by phosphorylated Akt were randomly sampled, from a set of feasible values, and the resulting trajectories were clustered according to a standard k-means algorithm, resulting in a finite set of possibilities. Details are provided in the Materials and Methods section.

Next, we attempted to connect PI3K-Akt signaling to the NF-κB pathway through the phosphorylation of IKK. We were unable to find an estimation of IKK activation rates in response to Akt. Therefore, to explore the possibilities, we conducted an extensive search of potential time-courses. Around 7000 simulations were run by randomly sampling feasible reaction rates, and the resulting NF-κB responses were grouped according to their characteristic trends (Figure 2).

### 3.3. Context Specificity and Validation

**Figure 3:**
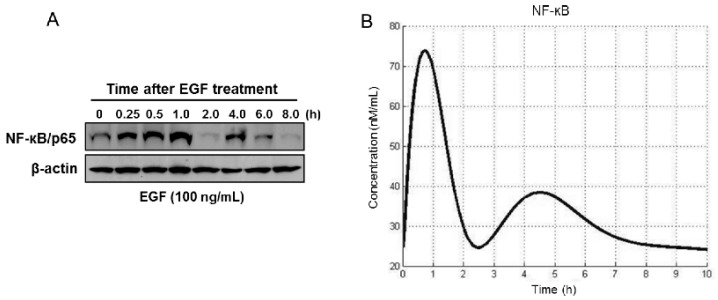
Simulation of constitutive activation of NF-κB response after EGF treatment. (**A**) RWPE1 cells were stimulated with EGF (100 ng/mL) for periods of 0.25 to 8 h and subjected to Western blotting for estimation of NF-κB/p65. (**B**) Computation of the time course of NF-κB activation. Details are provided in the Materials and Methods section.

In this experiment, RWPE1 cells were treated with EGF, solubilized with lysis buffer, and subjected to the immunoblot analysis using anti-NF-κB antibody. As shown in Figure 3, EGF induced NF-κB activation within 15 min, and the expression level was sustained for a subsequent 6 h. To recognize interactors within the NF-κB pathway, we selected a feasible set of parameters to reproduce the observed behavior of NF-κB-mediated EGF stimulation. For this, we explored possible categories of pathway behavior (defined by the reaction rates of IKK activation) and defined a set of model parameter values that qualitatively matched the available data in the literature to explore the categorical response of the model to observed mutations in prostate cancer.

**Figure 4:**
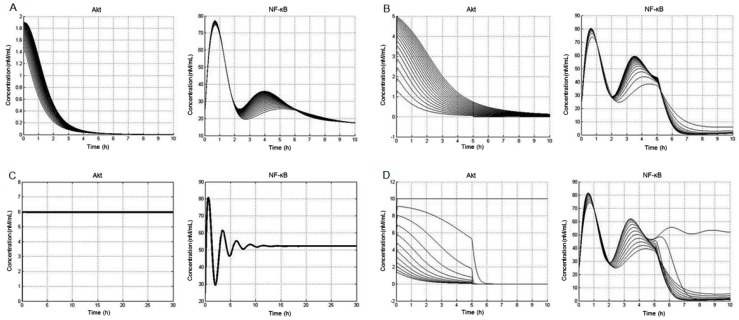
Computer simulation of the alteration in the PI3K-Akt pathway and its effects on Akt activation and nuclear NF-κB concentrations. In silico simulation of (**A**) epidermal growth factor receptor (EGFR) overexpression. The concentration of EGFR varied between 80 and 360 nM as the nominal value. The system was then stimulated with 360 nM EGF for 10 h. (**B**) PI3K overexpression. The concentration of PI3K varied between 10 and 100 nM as the nominal value. The system was then stimulated with 10 nM EGF for 5 h. (**C**) PTEN loss was modeled by eliminating the dephosphorylation of PIP3. The system was then stimulated with 10 nM EGF for 5 h. (**D**) PP2A mutation/loss was modeled by varying the concentration of PP2A between 0 and 11 nM as the nominal value. The system was then stimulated with 10 nM EGF for 5 h. Details are provided in the Materials and Methods section.

Next, we simulated responses that are affected by mutation of a particular gene(s), viz. PTEN or PP2A in the PI3K-Akt pathway, and studied their consequences on downstream NF-κB expression. To approach this, we first compiled a list of observed mutations within the PI3K-Akt pathway that have been reported in prostate cancer. These were then simulated to observe the effects on PI3K-Akt activation and nuclear NF-κB concentrations. As shown in Figure 4, in silico studies permit us to categorize mutations according to the severity of signaling pathway activity. These results suggest that overexpression of EGF receptors alone may not be sufficient to cause high levels of constitutive pathway activation and downstream NF-κB signaling (Figure 4A,B). On the contrary, PTEN loss (observed in >15% of prostate tumors) results in significantly higher levels of pathway activation that, surprisingly, persist even after the removal of EGF stimulation (Figure 4C). Moreover, mutation in PP2A and its loss might also affect NF-κB activation (Figure 4D). This narrows the broad range of suggested pathway abnormalities to those that most critically affect the pathway’s behavior.

### 3.4. EGF-Induced Cell Proliferation Increases NF-κB Activity and is Mediated by Increased Phosphorylation of Akt, IKK, and IκBα

**Figure 5:**
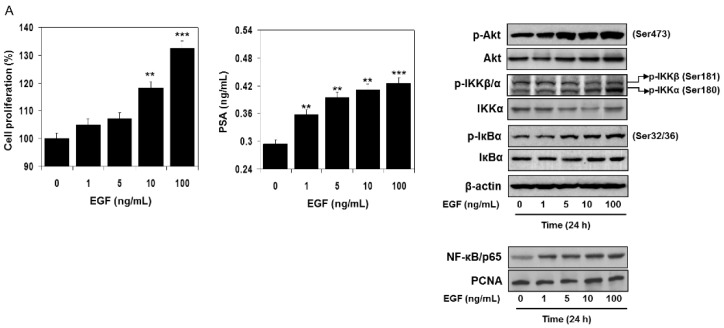

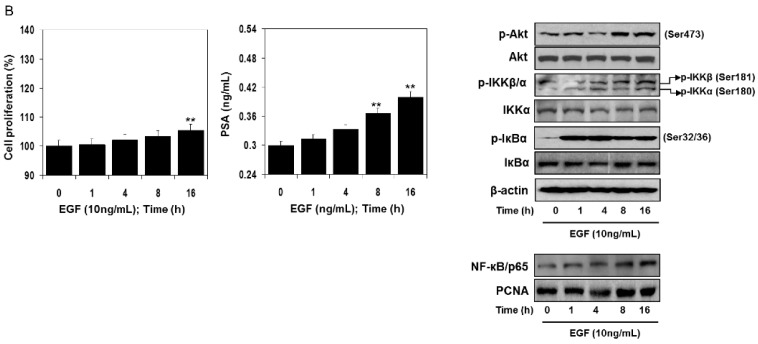
Effect of EGF treatment to RWPE1 cells in an (**A**) dose- and (**B**) time-dependent manner. RWPE1 cells were subjected to EGF exposure at doses between 1 to 100 ng/mL, and for time-dependent studies 10 ng/mL EGF was used. Cell proliferation was assessed by MTT assay and PSA by ELISA assay. Columns represent mean ± SD of three independent assays. ** *p* < 0.05 versus control. *** *p* < 0.001 versus control. Western blots for p-Akt (Ser473), Akt, p-IKKα/β, IKKα, p-IκBα, and IκBα in the total cell lysate and NF-κB/p65 in the nuclear fraction of RWPE1 cells after EGF exposure in a dose- and time-dependent manner. β-actin and PCNA were used as internal loading controls. Details are provided in the Materials and Methods section.

Next, we identified how EGF activates NF-κB. RWPE1 cells were stimulated with EGF and their cell extracts were prepared at different doses and times. Exposure of cells to EGF increased Akt phosphorylation at Ser473. An increase in the phosphorylation of IKK at Ser181/180 was noted post-EGF treatment, as such, p-IKKα levels were higher compared to p-IKKβ. Simultaneously, higher IκBα phosphorylation was observed in these cells following EGF exposure. These events led to subsequent activation and increased nuclear accumulation of NF-κB/p65, resulting in a higher proliferation rate and PSA secretion both in a dose- and time-dependent manner. No significant changes were noted in the protein levels of Akt, IKKα, and IκBα in the EGF-treated cell lysates (Figure 5A,B).

### 3.5. Akt Inhibition Decreases NF-κB Activation and Dependent Transcription

**Figure 6:**
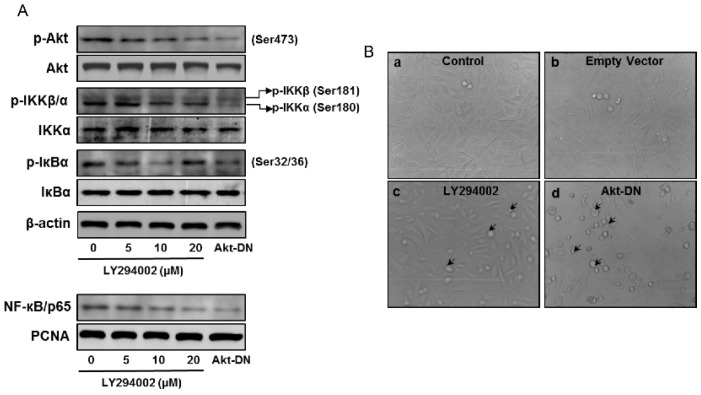
Effect of Akt inhibition on the expression of p-Akt (Ser473), Akt, p-IKKα/β, IKKα, p-IκBα, and IκBα in the total cell lysate and NF-κB/p65 in the nuclear fraction of RWPE1 cells. (**A**) Western blotting analysis in RWPE1 cells after treatment with Akt inhibitor, 5–20 µM LY294002, and transient knockdown of Akt using a dominant negative Akt (Akt DN) approach. β-actin and PCNA were used as internal loading controls. (**B**) Light microscopy of RWPE1 cells after treatment with LY294002 for 8 h, then Akt-DN vector and empty vector for 24 h. Arrows indicate cells undergoing apoptosis. Details are provided in the Materials and Methods section.

Akt is a critical component of signal transduction following PI3K activation. Two Akt inhibitors, LY294002 and Wortmanin, can efficiently block phosphorylation of Akt at T308 and S473 and inhibit Akt kinase activity. We transiently introduced an expression vector bearing dominant negative Akt (Akt DN) and an empty vector into RWPE1 cells, which resulted in downregulation of Akt. These approaches reduce the activating phosphorylation of Akt on Ser473 (Figure 6A). Treatment of RWPE1 cells with Akt inhibitor LY294002 and Akt DN reduced phosphorylation of IKK and IκBα and also reduced nuclear NF-κB levels. Inhibition in Akt activation led to reduced cell proliferation and increase apoptosis-like features in these cells (Figure 6B).

## 4. Discussion

In this study we provide the first steps toward computational analysis of mechanistic pathway components specific to the PI3K-Akt and NF-κB pathways perturbed in prostate cancer. We used a Bayesian data integration model to simultaneously provide information specific to biological contexts and individual biomolecular mechanisms for connecting these two signaling pathways during their activity in prostate-related biological contexts. Signaling between Akt and NF-κB is complex, however, the Akt pathway is actively involved in the regulation of NF-κB, and NF-κB activity is essential for oncogenic activity in prostate cancer. Studies conducted thus far in prostate cancer provide evidence that in cells with a gain of function in Akt due to loss of PTEN activity, the transcriptional activity of NF-κB is upregulated, and inhibition of Akt interferes with this upregulation of NF-κB [26,27,28]. Bai et al. have shown that chicken embryonic fibroblasts transformed by myristoylated Akt greatly enhance degradation of the IκB protein and increase phosphorylation of the NF-κB/p65 subunit [25]. These studies rule out the possibility of direct phosphorylation of the p65 or p50 NF-κB subunits by Akt. Furthermore, Akt phosphorylates IKKα, and the IKK complex not only targets the IκB inhibitor protein, but also phosphorylates the NF-κB/p65 subunit [37]. Thus, the IKK complex targets both the IκB and NF-κB/p65 proteins and functions as the intermediary between Akt and NF-κB. Additional studies are needed to validate these findings, at least in prostate cancer.

NF-κB is an important factor in the development and progression of cancer, in addition to being a central coordinator of the immune response [19,20,21]. The viral protein v-rel (homolog of c-rel) was originally identified as a retroviral oncogene. Several human tumors show constitutively elevated levels of NF-κB caused by genetic perturbation, including loss-of-function mutations in the IκB gene or activation of upstream regulators such as IKKs [38,39]. Blockade of NF-κB activity decreases tumorigenicity [40]. In prostate cancer, receptor activation occurs through signal transduction pathways involving tyrosine kinases, NF-κB-inducing kinase (NIK), and IKK, which ultimately leads to phosphorylation and faster turnover of IκBα, the super repressor of NF-κB activation [41]. NF-κB has also been shown to activate a transcription-regulatory element of the prostate-specific antigen (PSA)-encoding gene, a marker of prostate cancer development and progression [42]. These findings align with the present study in demonstrating an increase in PSA levels following NF-κB activation in virally transformed prostate epithelial cells. However, the importance of NF-κB activity for PI3K/Akt oncogenicity is particularly significant in view of the fact that the PI3K pathway is dysregulated in prostate cancer and other human malignancies. PI3K and Akt are considered promising cancer targets, and the dependence of these oncoproteins on NF-κB needs further evaluation.

A mathematical model that recapitulates TNF-induced IKK activity could be linked to the model of the NF-κB module and allow us to understand the dynamic control mechanisms in the context of prostate cancer [43]. For instance, Sizemore et al. showed that PI3K/Akt was necessary for the phosphorylation and activation of p65 in response to TNF and IL-1, and that Akt-mediated NF-κB activation requires IKK activity [44]. In contrast, Yang et al. reported that in mouse macrophages, LPS-induced p65 phosphorylation at S536 was unaffected by LY294002, an inhibitor of PI3K [45]. Ozes and colleagues found that Akt was an essential mediator of the TNFα-induced activation of NF-κB, operating through the phosphorylation of IKKα [46]. Similar studies will be insightful for other cytokine and pathogen receptor-associated signaling modules with an implicit prediction that the model of the IKK–IκB–NF-κB signaling module can be easily adapted to different cell types by measuring IκB mRNA and IKK activity time courses.

The dynamical properties of molecular biological systems are difficult to uncover by means of conventional molecular biology methods alone. A systems biology approach, on the other hand, enables us to generate and verify hypotheses concerning the unseen dynamical properties via interdisciplinary studies. From this point of view, a systems biology approach was utilized to investigate the convergence of highly anticipated signaling pathways, viz. PI3K-Akt and NF-κB, relevant to prostate cancer. The hypotheses were checked using the calibrated in silico model with further validation in an in vitro system. We were able to observe that only a few out of the broad range of suggested pathway abnormalities critically affected the pathway’s behavior significantly to cause constitutive activation of PI3K-Akt and NF-κB in prostate cancer. For instance, while over-expression of EGF receptors has been mentioned in the literature, in silico results suggest that this alone may not be sufficient to cause high levels of constitutive pathway activation. On the other hand, PTEN loss observed in >15% of prostate tumors results in significantly higher levels of pathway activation that persists even after the removal of EGF stimulation [31]. This systematic interrogation of the pathway behavior therefore suggests primary targets for future biological experimentation. Further studies are required to develop methodologies to connect various hierarchical multilevel pathways to discover prognostic indicators and their molecular signatures.

In conclusion, identification of the IKK complex as a coordination point in convergence of PI3K-Akt and NF-κB pathways in prostate cancer will serve as a guide for selection of biological experiments for the discovery of molecular drug targets. It is expected that interruption of this target may lead in the future design of therapeutics and/or strategies to improve current therapies. This new convergence identified from in silico experiments and validation in cell culture will be further investigated in its relationship to pathological conditions driving prostate cancer aggressiveness. 

## Supplementary Materials

The following are available online at https://www.mdpi.com/2073-4409/8/3/201/s1, Figure S1: The PI3K-Akt signaling pathway, Figure S2: The NF-κB signaling pathway.

## Abbreviations

Akt DN	dominant negative Akt
EGFR	epidermal growth factor receptor
FOXO	forkhead transcription factors
IĸBα	I-kappa-B-alpha
IKK	IκB kinase
NF-κB	Nuclear Factor-kappaB
ODE	ordinary differential equation
PDK	phosphotidylinositol-dependent kinase
PI3K	phosphatidylinositol 3′-OH kinase
PIP2	phosphoinositide 4,5-biphosphate
PIP3	phosphoinositide 3,4,5-triphosphate
PSA	prostate-specific antigen
PTEN	Phosphatase and tensin homolog
TNFα	tumor necrosis factor alpha
