# Supplementary figures and images for: Complex Systems Biology Approach in Connecting PI3K-Akt and NF-κB Pathways in Prostate Cancer
Source: Cells. 2019 Feb 26;8(3):201. doi: 10.3390/cells8030201 (PMC6468646; doi:10.3390/cells8030201)

Supplementary Figure 1

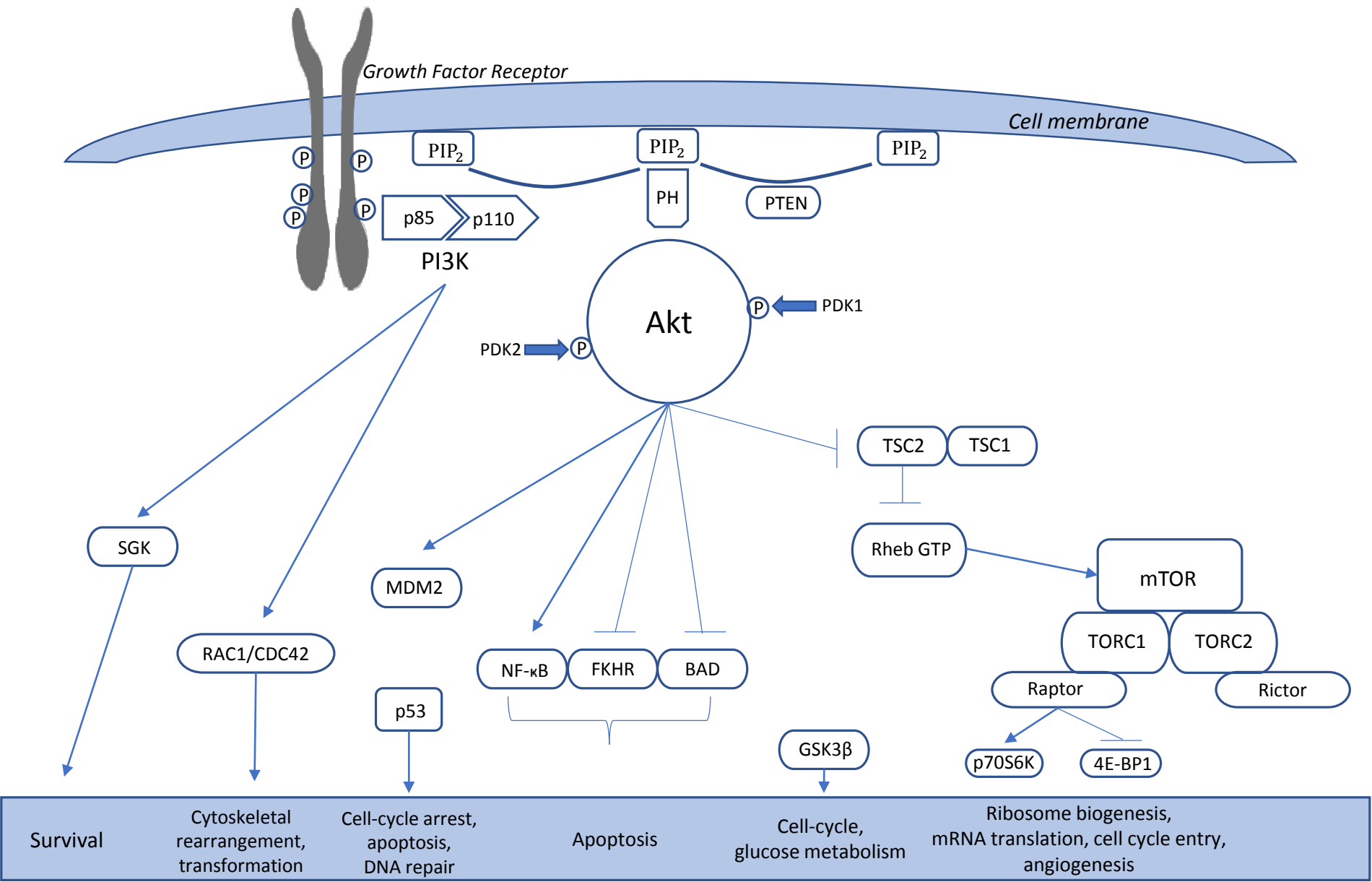

Supplementary Figure 2

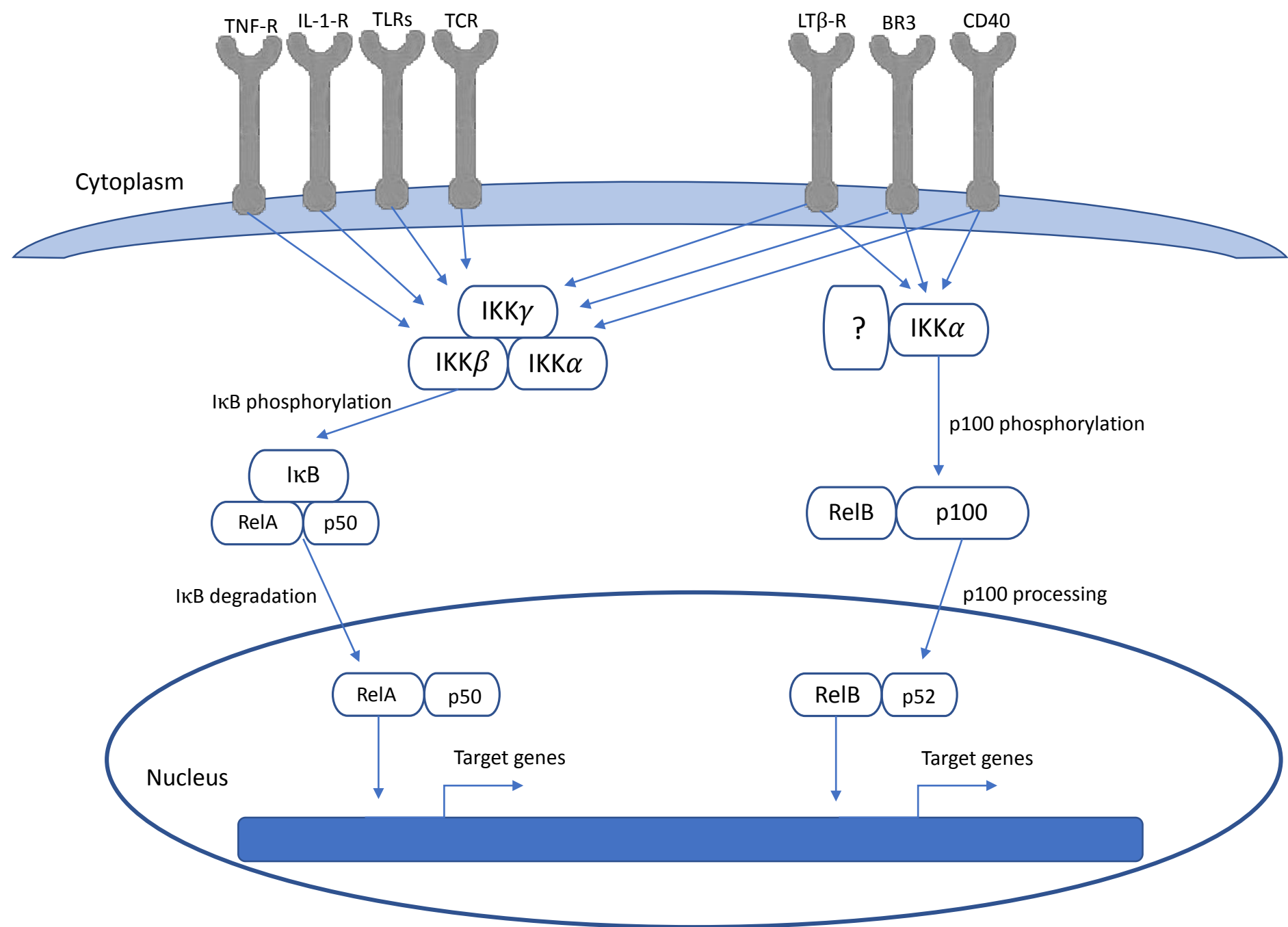

Supplement: Supplementary file 1 [file cells-08-00201-s001.pdf]
